# A novel multifunctional chiral metasurface with asymmetric transmission

**DOI:** 10.1038/s41598-024-76001-z

**Published:** 2024-10-21

**Authors:** Muhammad Noman, Hattan Abutarboush, Farooq A. Tahir, Adnan Zahid, Muhammad Imran, Qammer H. Abbasi

**Affiliations:** 1 James Watt School of Engineering, University of Glasgow, Glasgow, G12 8QQ UK; 2 College of Engineering, Taibah University, Madinah, Saudi Arabia; 3 School of Electrical Engineering and Computer Science (SEECS), National University of Sciences and Technology (NUST), Islamabad, Pakistan; 4 School of Engineering and Physical Science, Heriot Watt University, Edinburgh, EH14 4AS UK; 5 Artificial Intelligence Research Centre, Ajman University, Ajman, UAE

**Keywords:** Engineering, Electrical and electronic engineering

## Abstract

The multiband, multifunctional chiral metasurface with asymmetric transmission exhibits significant potential for diverse applications in modern communication systems, ranging from enhanced signal modulation and polarization control to advanced beam steering and compact antenna design. This research presents a versatile and advanced chiral metasurface operating at multiple bands with diverse functionalities, including asymmetric transmission. The proposed metasurface effectively transforms an incoming Linearly Polarized (LP) wave into a Circularly Polarized (CP) wave. Additionally, it functions as a 90° polarization rotator for the incident LP wave. The design starts with an element of a 2 × 2 supercell comprising a Square Split Ring Resonator (SSRR) and an I-shaped resonator. The right diagonal elements of a supercell undergo scaling down, giving rise to a rotational asymmetry. Chirality is introduced into the design, and cross polarization conversion is enhanced by rotating all four elements by 90° relative to each other. On the back side of the substrate, each element undergoes a 90° rotation compared to its counterpart on the front side, realizing the asymmetric transmission feature. The incorporation of multiband and multifunctional features within a single supercell equips the subject chiral metasurface to be utilized in various engineering applications.

## Introduction

The emergence of metasurfaces also identified as two-dimensional metasurfaces are artificial structures that have broken the natural material’s constraints to fully control and manipulate the propagating Electromagnetic (EM) waves. Parameters such as the polarization state, amplitude, and phase of EM waves are crucial for conveying vital information across various applications, spanning from microwave to optical frequencies^1–8^. Traditional techniques, however, are burdened by the drawbacks of requiring large and bulky structures, as well as limitations imposed by material resources. To address these challenges, chiral metasurfaces, a distinct subset lacking mirror symmetry in the direction of propagation, have been introduced. Leveraging strong chirality within their structure, chiral metasurfaces offer the capability to manipulate the polarization of EM waves effortlessly and arbitrarily. This characteristic gives rise to a range of compelling phenomena and applications, including chiral selective absorber^9,10^, cloaking^11,12^, lensing^13,14^, Negative Refractive Index (NRI)^15,16^, asymmetric transmission^17–21^, Circular Dichroism (CD: the absorption difference between RHCP and LHCP waves)^22^^-^^24^, polarization rotation or optical activity (LP to LP wave) and polarization conversion (LP to CP wave)^25–30^. Recently, multiband anisotropic metasurfaces are proposed to realize polarization conversion^31,32^. Besides, in addition to polarization conversion, specific EM functionalities such as beam generator, focusing, and hologram can be achieved by employing chiral metasurfaces or chiral structures^33–36^.

It is crucial to delve into the distinction between a polarization rotator and a converter at this juncture. In a polarization rotator, the outgoing EM wave maintains the same polarization state as the incoming wave, but the plane of polarization undergoes rotation by a certain angle. On the other hand, in a polarization converter, the polarization state shifts from a LP wave to a CP wave. Polarization converters find utility in various applications, such as controlling the polarization of radiating waves^37^ and reducing Radar Cross Section (RCS)^38^, etc. Chiral metasurface based polarization rotators and converters have enabled diverse manipulations of EM wave polarization, encompassing transformations from LP to LP, CP to CP, LP to CP. Numerous polarization rotators and converters, with singular and multiple functionalities, have been introduced across a spectrum from microwave to optical frequencies, encompassing both transmission and reflection types.

In recent times, the rapid progress in technology has led to an increased demand for multifunctional and multiband metasurfaces to address the intricate requirements of various applications. Some researchers have achieved multifunctional capabilities through active or anisotropic metasurfaces by modifying helicity^39^, polarization^40^, the direction of propagation of incident waves^41^ and employing external simulations^42^. While these efforts focus on reshaping wavefronts, the crucial and fundamental functionality of polarization manipulation, such as transforming LP wave to the other polarizations like CP wave, is seldom emphasized^43^. Chiral metasurfaces designed for polarization rotation and conversion are often composed of bilayer or multilayer structures. Previously introduced reconfigurable or tunable chiral metasurfaces^44–46^ typically necessitated additional control systems, leading to challenges in fabrication and concerns about reliability.

Given the challenges, constraints, and the increasing need for versatile multiband chiral metasurfaces across various applications, some researchers have recently devised passive chiral metasurfaces. In^47^ and ^48^, multiband and multifunctional chiral metasurface based polarization converters designed for transforming LP wave to CP wave, featuring asymmetric transmission. Another work, presented in^49^, exhibits a multiband Circular Polarized Selective (CPS) metasurface adept at converting LP to CP waves. Additionally, a multiband omega-shaped unit cell, highlighted in^50^, can convert LP to LP waves across five distinct frequency ranges, albeit limited to a single LP to CP polarization conversion. More recently, a multifunctional polarization converter utilizing a chiral metasurface is reported in^51^, demonstrating the conversion of LP to both CP and LP waves at two specific frequencies with asymmetric transmission. However, despite the high demand for multifunctional polarization control across various applications, no chiral metasurface design to date has achieved multiband integration for polarization conversion from LP to both RHCP and LHCP waves, as well as polarization rotation from LP to LP waves, all while incorporating asymmetric transmission.

Addressing these challenges and demands, this paper presents a multiband chiral metasurface that integrates various functionalities. The proposed 2 × 2 supercell consists of two distinct types of resonators with scaled-down right diagonal elements. Both front and backside elements of the substrate are rotated by 90° relative to each other. The utilization of a supercell with two resonator types aims to achieve robust chirality, high cross-polarized transmission, and effective polarization conversion and rotation by leveraging the mutual coupling inherent in the structure. The multiband and multifunctional chiral metasurface with asymmetric transmission effectively transforms the incident y-polarized wave into RHCP wave between 5.013‒5.066 GHz, and LHCP wave from 6.227‒6.297 GHz. For the incident x-polarized wave, it converts into an RHCP wave from 16.18‒16.285 GHz and rotates the x-polarized wave into a y-polarized wave within the frequency range of 14.988‒15.634 GHz. The performance of the designed chiral metasurface is validated through measurements after fabrication, demonstrating its versatility for numerous applications.

## Theory and design

### Theoretical analysis

**Fig. 1:**
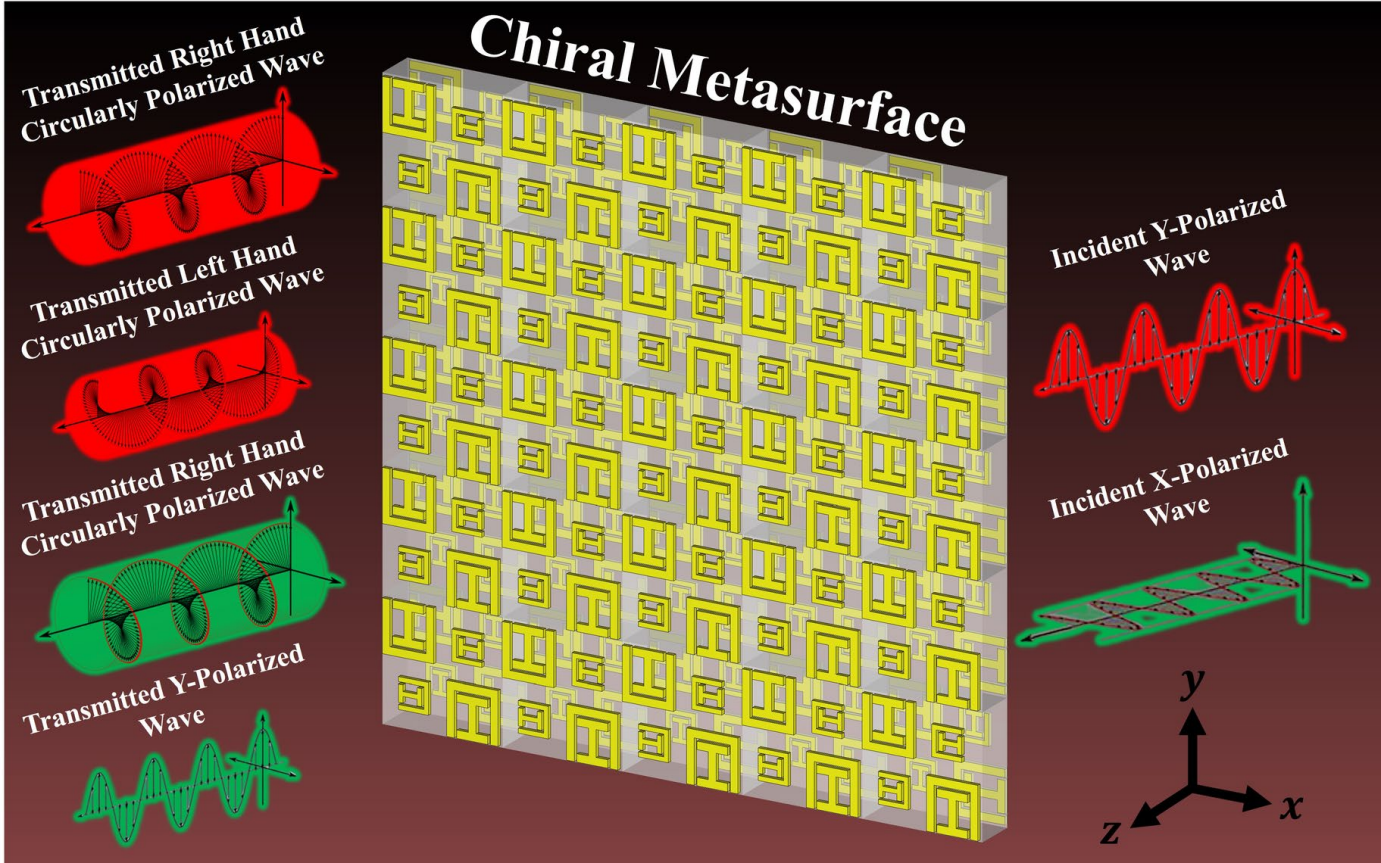
Schematic of a multiband and multifunctional chiral metasurface with asymmetric transmission which converts the LP wave into CP wave, and acts as a 90° polarization rotator.

For an intrinsic comprehension of the operational mechanism of the proposed multifunctional chiral metasurface based polarizer, a theoretical analysis is imperative. In this section, we delve into the theoretical analysis of the chiral metasurface depicted in Fig. 1, which not only converts LP wave into RHCP and LHCP wave but also functions as a 90° polarization rotator with asymmetric transmission. The design of the chiral metasurface involves an anisotropic structure, created through a supercell that incorporates two distinct subwavelength resonators arranged periodically on both the front and back sides. The constitutive relations governing the behavior of this structure are provided by^52^.

1 $$D={\epsilon }_{\circ }{\epsilon }_{r}E-\frac{jk}{{c}_{\circ }}H$$

2 $$B={\mu }_{\circ }{\mu }_{r}H+\frac{jk}{{c}_{\circ }}E$$

Here, the symbols k, ${c}_{\circ }$, ${\epsilon }_{\circ }$(${\epsilon }_{r}$) and ${\mu }_{\circ }$(${\mu }_{r}$) represent chirality, describing the strength of cross coupling between the electric and magnetic fields, the speed of light in free space, permittivity, and permeability in free space (relative), respectively. Let’s consider an incident plane wave propagating in the forward direction (+ z). The incident electric field ${E}^{inc}$ and transmitted electric field ${E}^{tr}$ can be defined as [53]:

3 $${E}^{inc}\left(x, y, z, t\right)=\left(\genfrac{}{}{0pt}{}{{E}_{x}^{inc}}{{E}_{y}^{inc}}\right){e}^{i(kz-\omega t)}$$

4 $${E}^{tr}\left(x, y, z, t\right)=\left(\genfrac{}{}{0pt}{}{{E}_{x}^{tr}}{{E}_{y}^{tr}}\right){e}^{i(kz-\omega t)}$$

Here, ${E}_{x}$, ${E}_{y}$, k and ω represent the complex amplitudes of the x- and y-components of an electric field, wave number, and frequency, respectively. To enhance understanding of the proposed chiral metasurface based polarizer, we define the Transmission Component (TC) and transmission matrix for the LP wave as follows:

5 $$Transmission Component (TC)=\frac{{E}^{tr}}{{E}^{inc}}$$

6 $$\left(\genfrac{}{}{0pt}{}{{E}_{x}^{tr}}{{E}_{y}^{tr}}\right)={(TC)}_{Linear}\left(\genfrac{}{}{0pt}{}{{E}_{x}^{inc}}{{E}_{y}^{inc}}\right)=\left(\genfrac{}{}{0pt}{}{{T}_{xx}}{{T}_{yx}}\genfrac{}{}{0pt}{}{{T}_{xy}}{{T}_{yy}}\right)\left(\genfrac{}{}{0pt}{}{{E}_{x}^{inc}}{{E}_{y}^{inc}}\right)$$

Therefore, the transmitted CP wave can be defined as:

7 $$\left(\genfrac{}{}{0pt}{}{{E}_{+}^{tr}}{{E}_{-}^{tr}}\right)=\left(\genfrac{}{}{0pt}{}{{E}_{x}^{tr}}{{E}_{x}^{tr}}\genfrac{}{}{0pt}{}{{+jE}_{y}^{tr}}{{-jE}_{y}^{tr}}\right)={(TC)}_{Circular}\left(\genfrac{}{}{0pt}{}{{E}_{x}^{inc}}{{E}_{y}^{inc}}\right)$$

8 $${(TC)}_{Circular}=\left(\genfrac{}{}{0pt}{}{{T}_{+x}}{{T}_{-x}}\genfrac{}{}{0pt}{}{{T}_{+y}}{{T}_{-y}}\right)=\frac{1}{\sqrt{2}}\left(\genfrac{}{}{0pt}{}{{T}_{xx}{+jT}_{yx}}{{T}_{xx}{-jT}_{yx}}\genfrac{}{}{0pt}{}{{T}_{xy}{+jT}_{yy}}{{T}_{xy}{-jT}_{yy}}\right)$$

Here, the symbols “ + ” and “–” denote the RHCP and LHCP transmitted waves, respectively. The inclusion of the factor $\frac{1}{\sqrt{2}}$ is for power normalization.

### Design of chiral metasurface

**Fig. 2:**
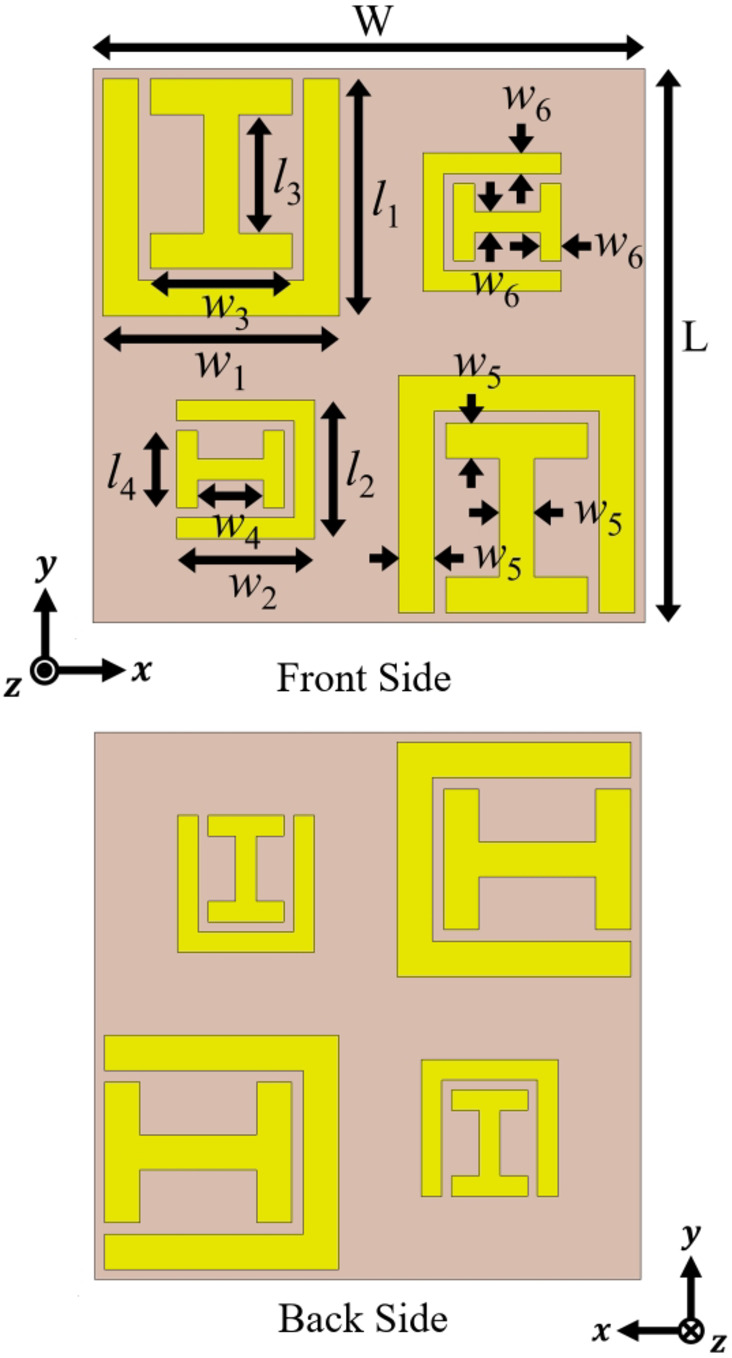
Schematic and geometric dimensions of the proposed chiral metasurface supercell.

The diagram in Fig. 1 illustrates a proposed bilayer chiral metasurface with multiband and multifunctional capabilities. When subjected to illumination by LP plane wave, this metasurface transforms the transmitted wave into both RHCP and LHCP states. Additionally, it functions as a 90° polarization rotator, exhibiting an asymmetric response across four distinct frequency bands. The design process for the chiral metasurface starts with an element of a 2 × 2 supercell placed on a cost-effective FR-4 substrate with material properties: a relative permittivity ${\epsilon }_{r}=4.3$, a loss tangent $\delta =0.025$, and a thickness of 1.6 mm. This element incorporates two distinct subwavelength resonators, SSRR and an I-shaped resonator. An element is duplicated periodically on the front side, each repetition involving a 90° clockwise rotation, forming a 2 × 2 supercell. After that, the right diagonal elements are scaled down in comparison to the left diagonal elements, deliberately breaking the corner-four (C4) rotational symmetry of the structure. This asymmetry serves to enhance cross polarization conversion. The geometry of the proposed chiral metasurface supercell with all the dimensions is shown in Fig. 2.

Due to the lack of C4 symmetry in the structure, the transmission components of the LP plane wave exhibit characteristics where ${T}_{xx}^{f(b)}={T}_{yy}^{f(b)}$ and ${T}_{yx}^{f(b)}\ne {T}_{xy}^{f\left(b\right)}, {T}_{yx}^{f(b)}={T}_{xy}^{b(f)}$. The elements are positioned on the back side of the substrate after a 90° rotation in comparison to the elements on the front side. This arrangement is implemented to establish strong chirality within the structure. The final optimized parameters for the chiral metasurface supercell can be found in Table 1.

**Table 1:** Optimized parameters of chiral metasurface supercell

Parameter	Value (mm)	Parameter	Value (mm)	Parameter	Value (mm)
L, W	14	***l***_**3**_	3	***w***_**4**_	1.675
*l*_1_, *w*_1_	6	***l***_**4**_	1.95	***w***_**5**_	0.9
*l*_2_, *w*_2_	3.5	***w***_**3**_	3.6	***w***_**6**_	0.525

The proficiency of any polarizer based on a chiral metasurface in converting the incident LP wave into transmitted RHCP and LHCP waves can be observed through the application of Eq. (8). Subsequently, the performance can be assessed using various parameters such as Polarization Extinction Ratio (PER), ellipticity angle (η), Magnitude and Phase Difference (MD & PD), and Circular Dichroism (CD). Additionally, for a polarizer to achieve the rotation of the incident LP plane wave into cross polarization, it is essential to evaluate parameters such as Polarization Conversion Ratio (PCR) and azimuth angle (θ) [30,49].

9 $$\text{PER}=20\text{log}10\left(\frac{\left|{T}_{+x(y)}\right|}{\left|{T}_{-x(y)}\right|}\right)$$

10 $$\eta =\text{arctan}\left(\frac{\left|{T}_{+x(y)}\right|-\left|{T}_{-x(y)}\right|}{\left|{T}_{+x(y)}\right|+\left|{T}_{-x(y)}\right|}\right)=0.5\text{arcsin}\left(\frac{{\left|{T}_{+x(y)}\right|}^{2}-{\left|{T}_{-x(y)}\right|}^{2}}{{\left|{T}_{+x(y)}\right|}^{2}+{\left|{T}_{-x(y)}\right|}^{2}}\right)$$

11 $$\text{MD}=\left(\frac{{T}_{yx(xy)}}{{T}_{xx(yy)}}\right)$$

12 $$\text{PD}=\text{arg}({T}_{yx\left(xy\right)})-({T}_{xx(yy)})$$

13 $$\text{CD}=\left|{T}_{+x(y)}\right|-\left|{T}_{-x(y)}\right|$$

14 $$\text{PCR}=\left(\frac{{\left|{T}_{xy(yx)}\right|}^{2}}{{{\left|{T}_{yy(xx)}\right|}^{2}+\left|{T}_{xy(yx)}\right|}^{2}}\right)$$

15 $$\theta =\left(\frac{\text{arg}({T}_{+x(y)})-\text{arg}({T}_{-x(y)})}{2}\right)$$

The determination of the transmitted wave as RHCP or LHCP is based on certain criteria. Specifically, when PER ≥  ± 20 dB, η =  ± 45°, MD = 1, and PD =  ± 90°, the transmitted wave is considered to be RHCP or LHCP. A higher value of PER indicates a more efficient polarization conversion. The ideal scenario for η is ± 45°, signifying the transmitted wave as a CP wave. In practical cases, the value of η is typically around ± 45°. Furthermore, for a transmitted wave to undergo a 90° polarization rotation, PCR needs to be ≥ 0.9, and θ =  ± 90°.

## Results and discussion

**Fig. 3:**
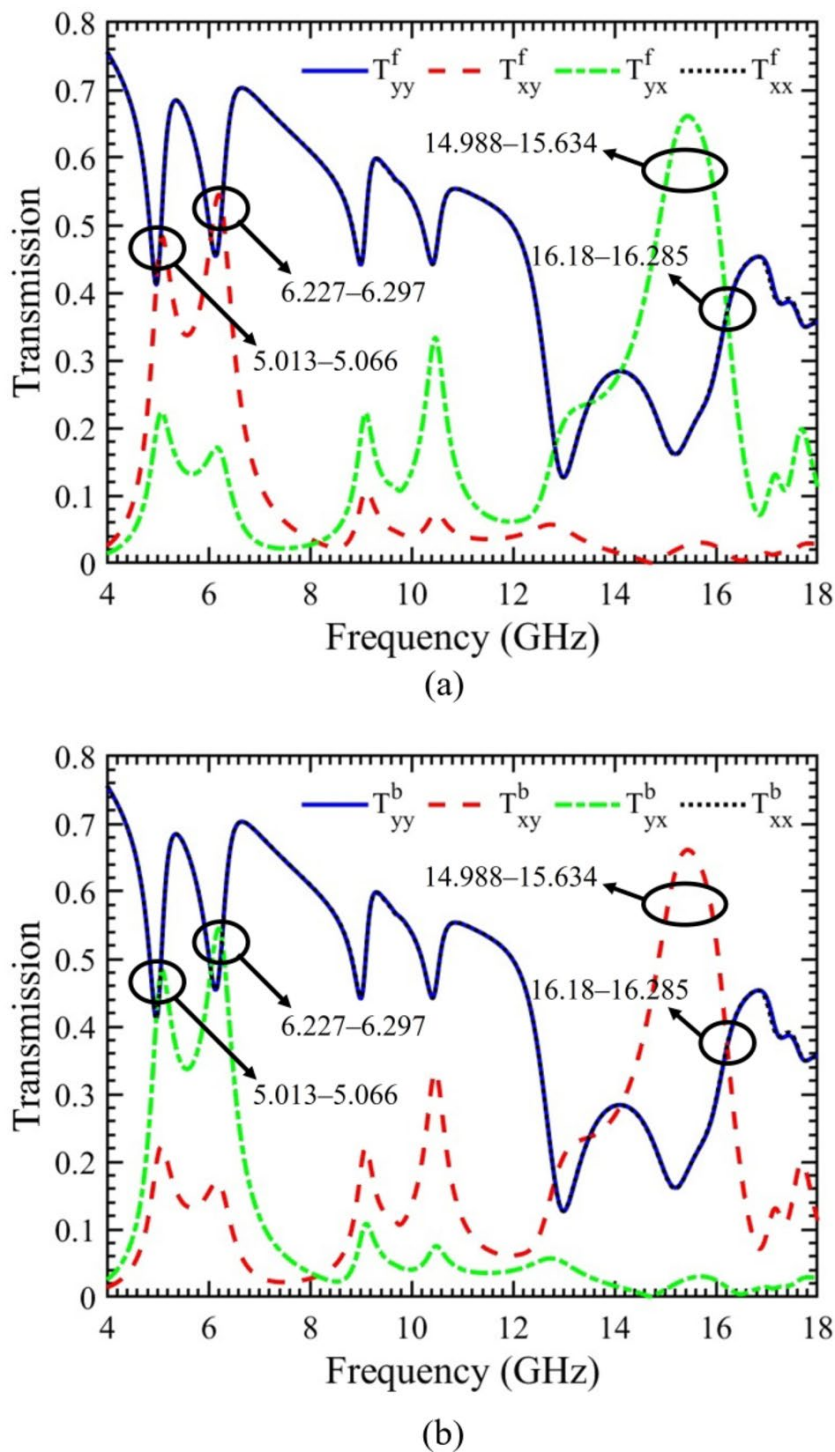
Magnitude of the transmission components of x- and y-polarized incident waves. (**a**) Forward propagation. (**b**) Backward propagation.

A linearly polarized plane wave is directed towards the proposed chiral metasurface to investigate and understand the behavior of the structure. The simulated magnitudes of the transmitted components for both x- and y-polarized waves, propagating in the forward (+ z) and backward (− z) directions as a function of frequency, are illustrated in Fig. 3(a & b), respectively. For the y-polarized incident wave propagating in the forward direction, the transmission components magnitudes are depicted in Fig. 3(a). Notably, two distinct frequency bands, namely 5.013‒5.066 GHz and 6.227‒6.297 GHz. In these bands, the magnitudes of the transmission co-component $(\left|{T}_{yy}\right|)$ and cross-component $(\left|{T}_{xy}\right|)$ are nearly identical. Consequently, it can be inferred that a complete conversion from LP to CP wave occurs at these specific frequency bands. When an incident wave with x-polarization propagates in the forward direction within the frequency range of 16.18‒16.285 GHz, there is a strong conversion from LP to CP wave. This inference is drawn from the observation that the magnitudes of the transmission co-component $(\left|{T}_{xx}\right|)$ and cross-component

$(\left|{T}_{yx}\right|)$ are nearly identical during this frequency band. It is crucial to highlight that, during these three frequency bands, the magnitudes of the co-components $(\left|{T}_{yy}\right|)$ and $(\left|{T}_{xx}\right|)$ are equal, achieving one of the fundamental conditions for the creation of LP to CP waves.

Moreover, when an incident wave is x-polarized and propagates in the forward direction, a pronounced and wideband polarization rotation is evident within the frequency range of 14.988‒15.634 GHz. In this frequency band, the magnitude of the transmission cross-component $(\left|{T}_{yx}\right|)$ reaches its peak value, while the co-component $(\left|{T}_{xx}\right|)$ attains its lowest magnitude. Consequently, a wideband cross-polarization from x- to y-polarized wave is achieved. The simulated magnitudes of transmission components for x- and y-polarized waves, propagating in backward (− z) direction as a function of frequency are illustrated in Fig. 3(b). This graph serves to highlight the asymmetric transmission response of the structure. As previously mentioned, the proposed chiral metasurface lacks C4 symmetry. Consequently, while the response of co-components remains consistent, the response of cross-components changes, as indicated by ${T}_{xx}^{f(b)}={T}_{yy}^{f(b)}$ and ${T}_{yx}^{f(b)}\ne {T}_{xy}^{f\left(b\right)}, {T}_{yx}^{f(b)}={T}_{xy}^{b(f)}$.

### Polarization extinction ratio and ellipticity

**Fig. 4:**
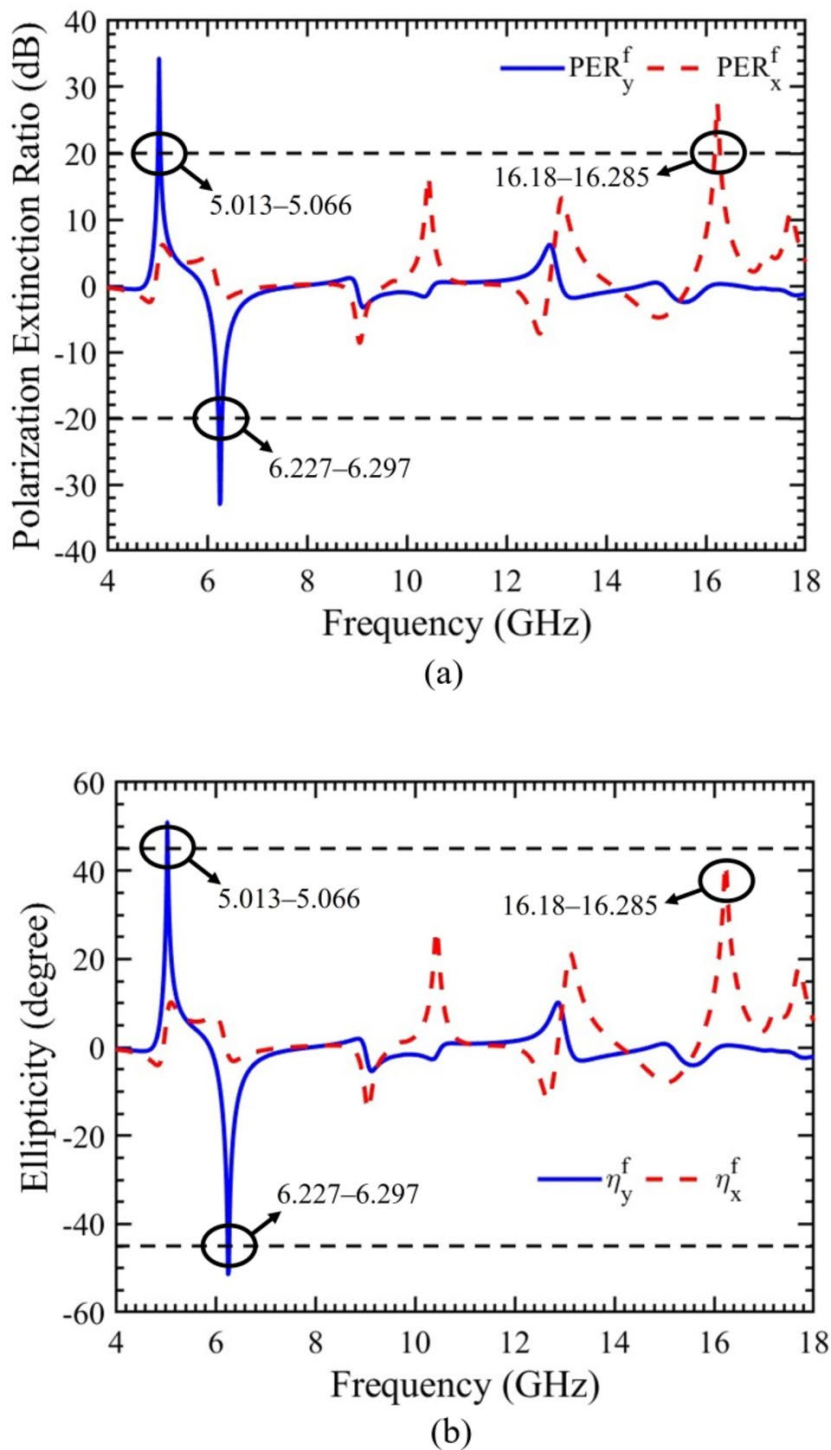
For x- and y-polarized incident waves propagating in forward direction. (**a**) Polarization extinction ratio. (**b**) Ellipticity angle.

The chiral metasurface’s capability to convert LP wave into CP wave and LP to LP can be assessed using various performance parameters and criteria defined in Eqs. (9–15). Among these parameters, the PER serves as a key metric, illustrating the attainment of circular polarization across different frequencies. The calculated PER values for x- and y-polarized incident waves propagating in the forward direction are presented in Fig. 4(a). Specifically, for the y-polarized wave, (PER_y_) is >  + 20 dB within the frequency range of 5.013‒5.066 GHz and >  − 20 dB within the 6.227‒6.297 GHz frequency band, as indicated by horizontal dashed lines. This implies that the incident y-polarized wave is effectively converted into RHCP and LHCP waves, respectively. Similarly, for the x-polarized wave, (PER_x_) is >  + 20 dB within the 16.18‒16.285 GHz frequency range, indicating successful conversion from LP to RHCP wave. Notably, for all three frequency bands where the conversion from LP to CP wave occurs, the PER values are significantly above ± 20 dB, signifying complete LP-CP polarization conversion.

To further confirm the polarization conversion at the aforementioned three frequency bands, the ellipticity angle is computed, as illustrated in Fig. 4(b). Specifically, for the y-polarized wave, the calculated value of (η_y_) is >  ± 45° within the frequency range of 5.013‒5.066 GHz and 6.227‒6.297 GHz, as indicated by horizontal dashed lines. This signifies a pure transmitted conversion from LP to CP wave. For the x-polarized wave, the calculated value of (η_x_) within the frequency band of 16.18‒16.285 GHz is closely approaching + 45°, indicating the transmission of a CP wave. This further supports and validates the successful conversion from LP to CP wave at the specified frequency bands.

### Magnitude and phase difference

**Fig. 5:**
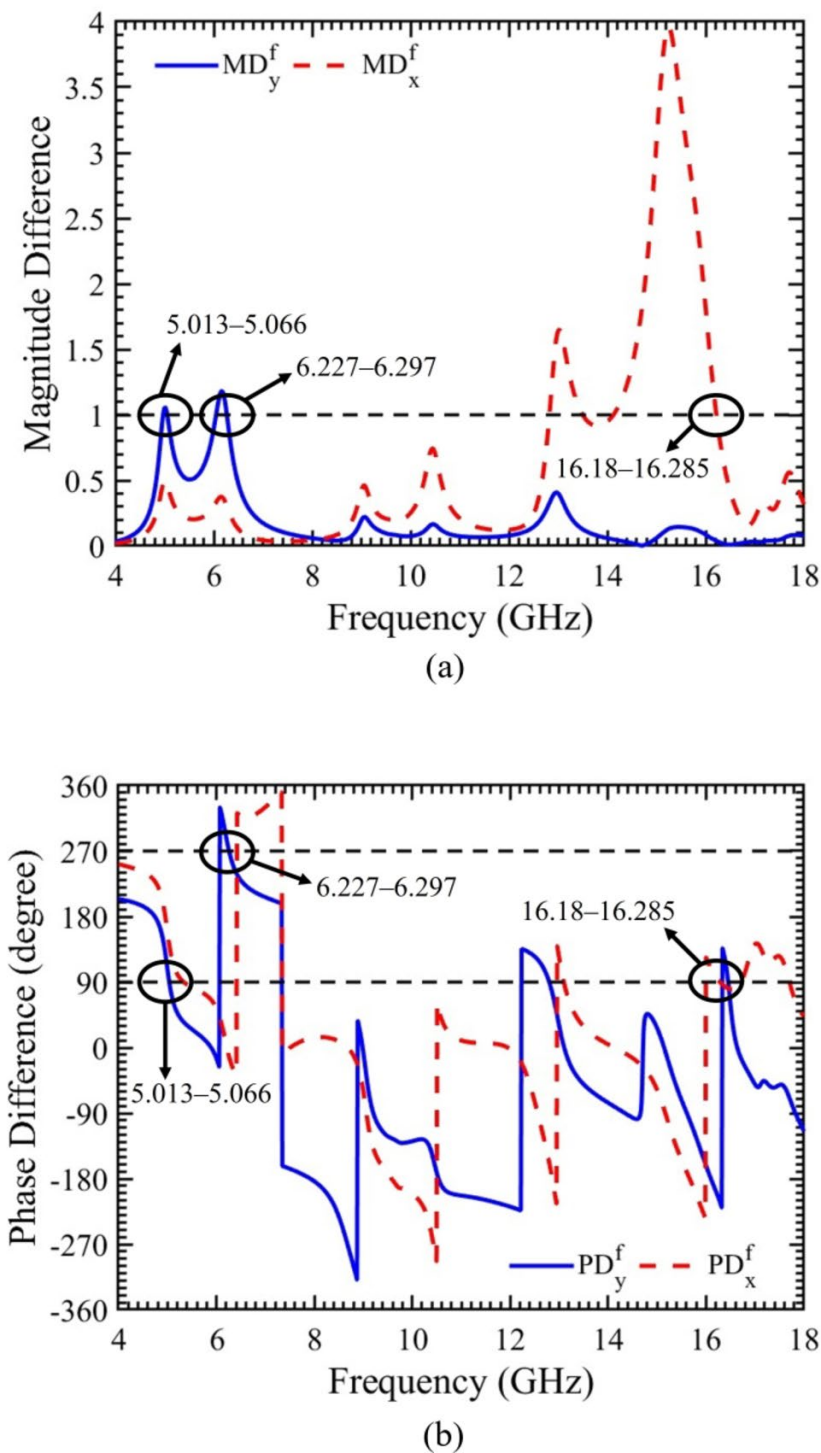
For x- and y-polarized incident waves propagating in forward direction. (**a**) Magnitude difference. (**b**) Phase difference.

To further illustrate the conversion from LP to CP waves at three distinct frequency bands (5.013‒5.066 GHz, 6.227‒6.297 GHz, and 16.18‒16.285 GHz), the magnitude and phase differences for x- and y-polarized waves propagating in the forward direction are computed, as depicted in Fig. 5(a & b). In Fig. 5(a), it is evident that the magnitude differences for both x- and y-polarized waves (MD_y_ & MD_x_) closely approaching “1” across all three frequency bands, as indicated by horizontal dashed line.

Additionally, the phase differences at the three specified frequency bands are calculated and presented in Fig. 5(b). For the y-polarized wave propagating in the forward direction, the phase difference (PD_y_) is nearly + 90° within the 5.013‒5.066 GHz frequency band and approximately two times a multiple of + 90° within the 6.227‒6.297 GHz frequency band, as indicated by horizontal dashed lines. For the x-polarized wave, the phase difference (PD_x_) within the 16.18‒16.285 GHz frequency band is close to + 90°. Assessing both magnitude and phase differences to evaluate the polarization conversion ability at the aforementioned frequency bands, it can be concluded that a robust and efficient conversion from LP to RHCP and LHCP waves is achieved.

### Linear to circular transmission components and circular dichroism

**Fig. 6:**
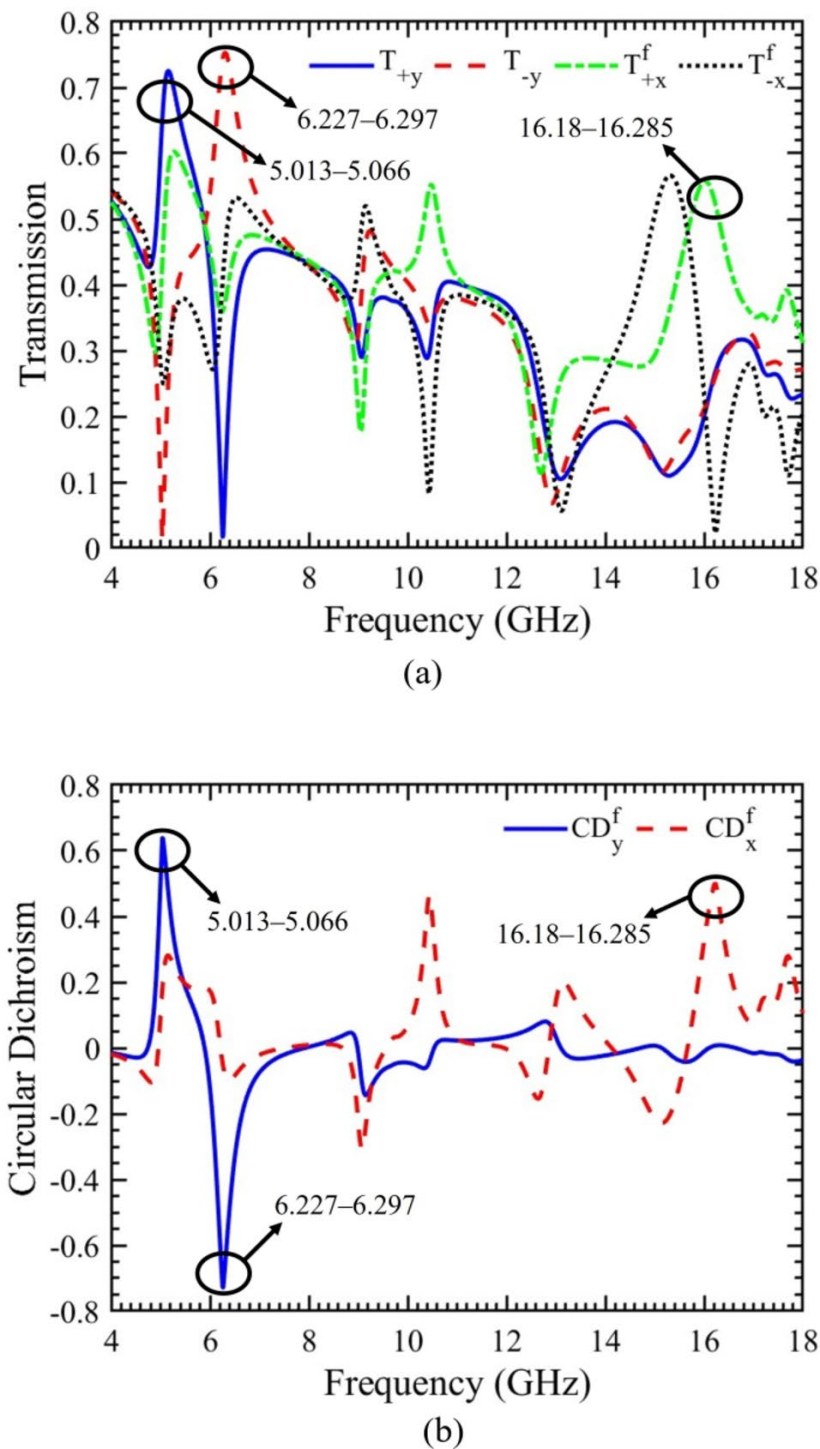
For x- and y-polarized incident waves propagating in forward direction. (**a**) Linear to circular transmission components. (**b**) Circular dichroism.

The proposed chiral metasurface effectively transforms the incident LP wave into RHCP or LHCP waves as represented by CD, a distinctive property of chiral metasurfaces. To achieve this, the linear transmission components ${(TC)}_{Linear}$ are converted into circular transmission components ${(TC)}_{Circular}$. Subsequently, the linear to circular transmission components are determined using Eq. 8. The magnitude of linear to circular transmission components for x- and y-polarized incident waves propagating in the forward direction is presented in Fig. 6(a). In the case of the y-polarized incident wave, the circular transmission component for RHCP waves (T_+y_) surpasses the value for LHCP wave (T_−y_) within the frequency range of 5.013‒5.066 GHz. Conversely, it exhibits a lower value for RHCP wave compared to LHCP wave within the 6.227‒6.297 GHz frequency band. This observation indicates a robust polarization conversion from LP to RHCP and LHCP waves for 5.013‒5.066 GHz and 6.227‒6.297 GHz, respectively. Similarly, for the x-polarized incident wave, the circular transmission component for RHCP wave (T_+x_) surpasses the value for LHCP wave (T_−x_) within the frequency range of 16.18‒16.285 GHz. This confirms the realization of a strong RHCP wave.

Using Eq. 13 and based on the linear to circular transmission components, a significant property of the chiral metasurface known as CD is calculated, as illustrated in Fig. 6(b). CD refers to the difference in absorption between RHCP and LHCP waves, whether in transmission or reflection mode. The results in Fig. 6(b) affirm that, for the y-polarized incident wave, the proposed chiral metasurface allows the passage of RHCP and LHCP waves while impeding LHCP and RHCP waves for the frequency ranges of 5.013‒5.066 GHz and 6.227‒6.297 GHz, respectively. Consequently, a pronounced circular dichroism effect (CD_y_) is achieved at these frequency bands. Similarly, for the x-polarized incident wave, the metasurface allows the transmission of RHCP wave while inhibiting LHCP wave within the frequency range of 16.18‒16.285 GHz, resulting in a robust circular dichroism effect (CD_x_).

### Polarization conversion ratio and azimuth angle

**Fig. 7:**
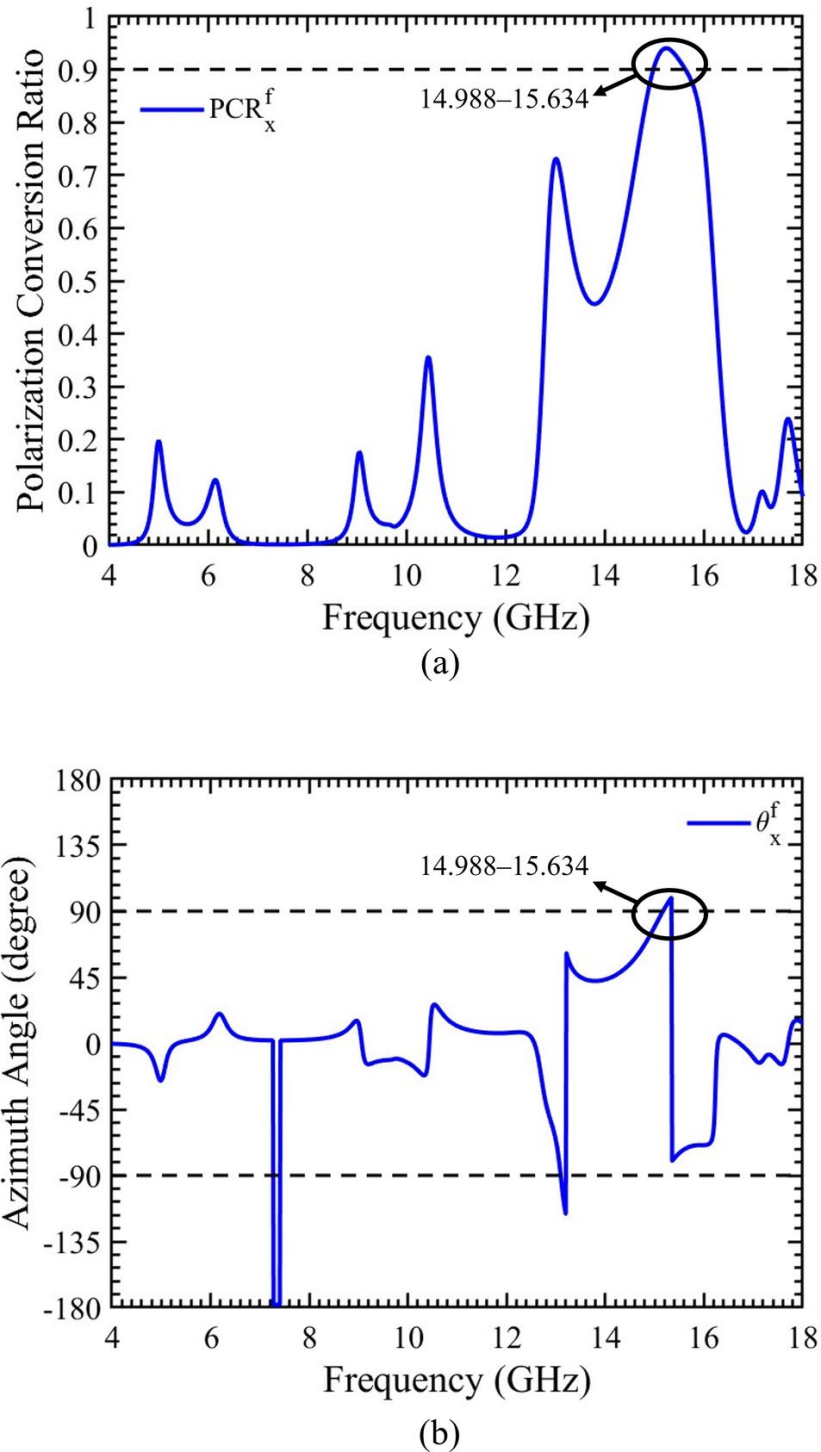
For x-polarized incident waves propagating in forward direction. (**a**) Polarization conversion ratio. (**b**) Azimuth angle.

To further validate the multifunctional capabilities of the proposed chiral metasurface, particularly its role as a polarization rotator, two key parameters, namely PCR and azimuth angle, are calculated and depicted in Fig. 7(a & b). For an x-polarized incident wave propagating in the forward direction, the metasurface effectively functions as a 90° polarization rotator within the frequency band of 14.988‒15.634 GHz. This is evidenced by the value of PCR (PCR_x_) exceeding “0.9,” as indicated by horizontal dashed line in Fig. 7(a). Simultaneously, the azimuth angle (*θ*_x_) for the specified frequency band remains within the range of ± 90°, as illustrated in Fig. 7(b). These results collectively demonstrate the metasurface’s capability to achieve a wideband LP to LP cross polarization, converting x-polarized wave into y-polarized wave for the frequency range of 14.988‒15.634 GHz.

### Surface current distribution

The polarization conversion behavior of the proposed supercell structure is explained through an analysis of the surface current distribution. At resonant frequencies, polarization conversion or significant cross-polarization transmission occurs, driven by both longitudinal dipole–dipole interactions and transverse (interlayer) coupling. The combined effect of modes generated by magnetic and electric dipoles is represented by the eigenmodes of the resonator.

**Fig. 8:**
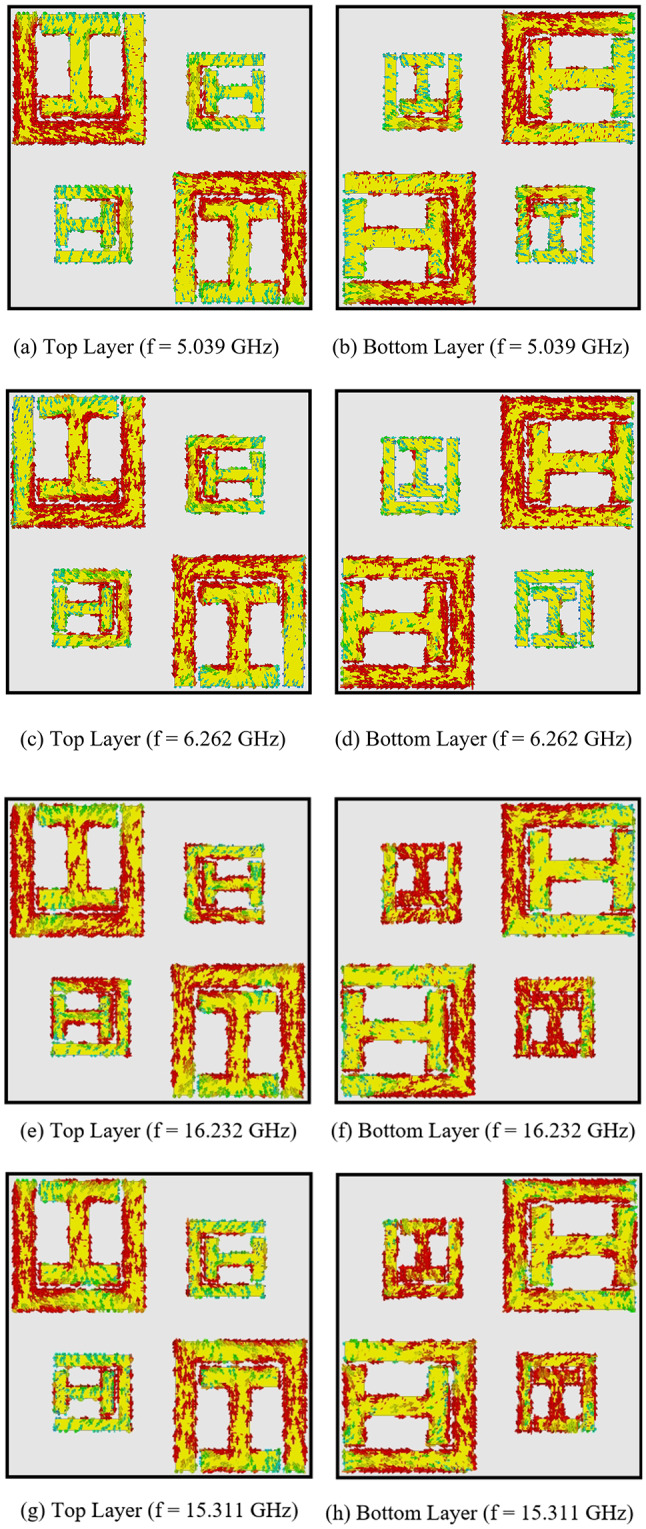
Surface current distribution on the top and bottom layers of proposed metasurface at different frequencies.

Figure 8 illustrates the surface current distribution at different resonant frequencies. At 5.039 GHz, a longitudinal electric dipole mode is observed in the two SSRR elements on the left diagonal of the top layer. This shows weak coupling within the supercell but strong coupling with neighboring supercell elements. Meanwhile, a magnetic dipole mode appears in the right diagonal elements due to their small size, as shown in Fig. 8(a). In Fig. 8(b), the bottom layer reveals reversed currents in the left diagonal elements and parallel magnetic dipole coupling in the right diagonal elements. This difference in rotation direction determines whether the transmitted wave is RHCP or LHCP. At 5.039 GHz, the parallel currents on both layers indicate an RHCP wave.

At 6.262 GHz, the current distribution on the top layer resembles that at 5.039 GHz, particularly in the right diagonal elements, as shown in Fig. 8(c). However, Fig. 8(d) shows antiparallel currents in the right diagonal elements on the bottom layer compared to the top, resulting in an LHCP wave. Similarly, at 16.232 GHz, the parallel currents across both layers result in an RHCP wave, as depicted in Fig. 8(e, f).

Utilizing different resonators within the unit cell enables strong coupling across multiple regions, offering flexibility in controlling the electromagnetic behavior of the structure. At 15.311 GHz, strong cross-polarization conversion is evident, and the field distributions become twisted due to the antiparallel current flow, as shown in Fig. 8(g, h).

## Fabrication and measurement

**Fig. 9:**
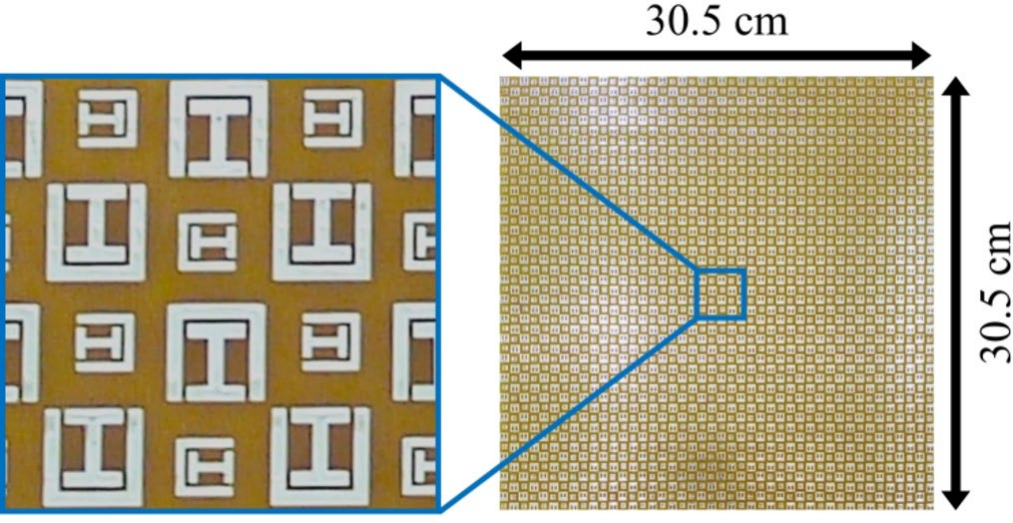
Photograph of the fabricated chiral metasurface.

**Fig. 10:**
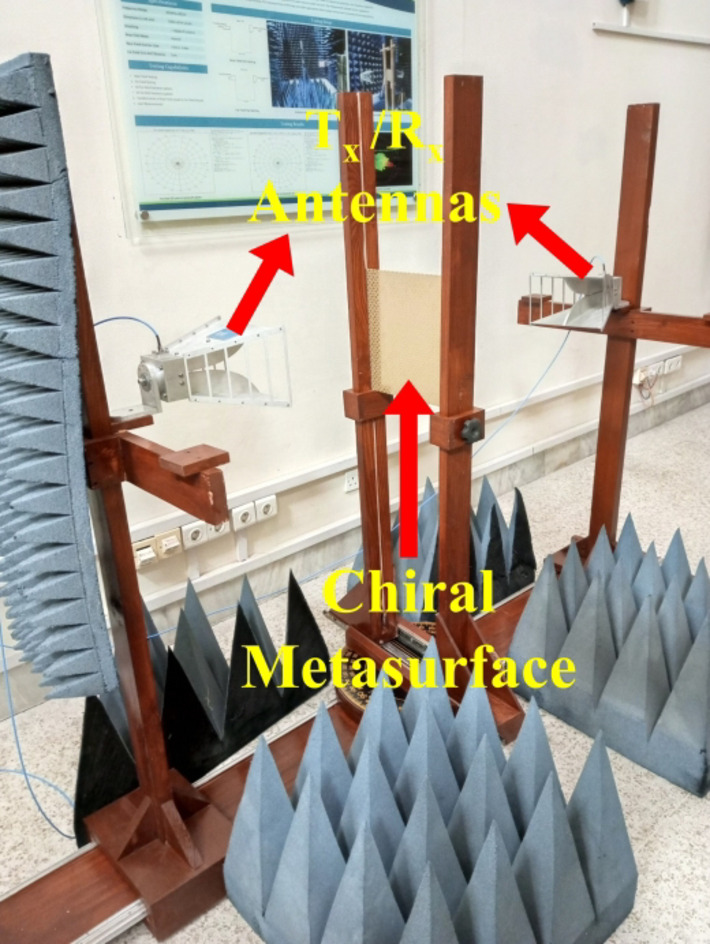
Photograph of the measurement setup.

**Fig. 11:**
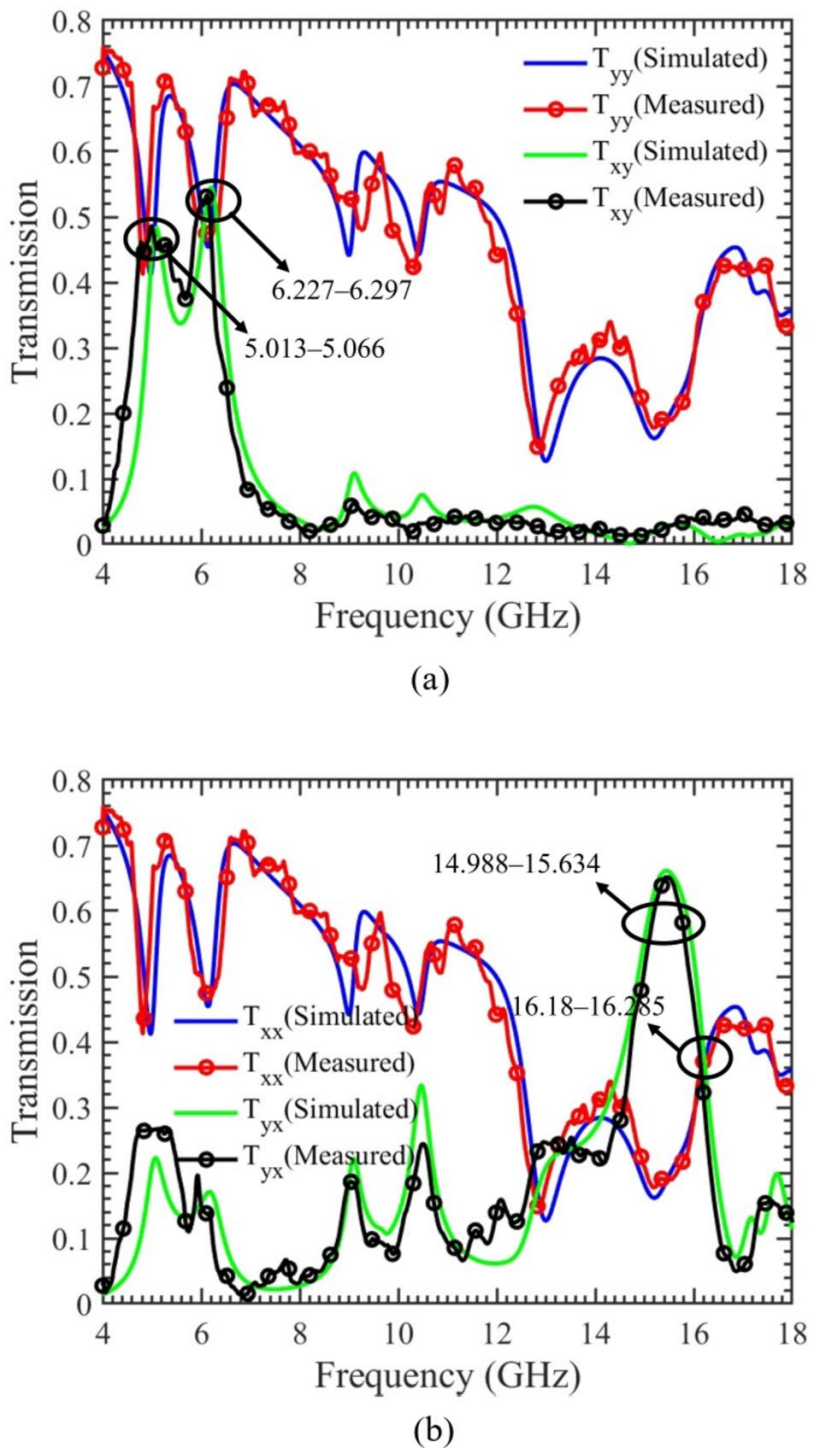
Simulated and measured magnitude of the transmission components propagating in the forward and backward direction. (**a**) y-polarized incident waves. (**b**) x-polarized incident waves.

The chiral metasurface proposed in this study was fabricated through a standard Printed Circuit Board (PCB) process. A visual representation of the fabricated prototype is provided in Fig. 9. To assess and validate the performance of the proposed chiral metasurface, experimental measurements were conducted in the lab, within an open environment. The experimental setup involved the use of two wideband horn antennas for transmitting and receiving EM waves. The chiral metasurface was positioned between the horn antennas and surrounded by pyramidal absorbing material, as depicted in Fig. 10. The antennas were connected to a Vector Network Analyzer (VNA) model E8362B using coaxial cables. To measure the magnitude of transmission co-component ($\left|{T}_{xx}\right|$) and ($\left|{T}_{yy}\right|$), transmitting and receiving antennas were positioned in a co-polarized position, aligning either in horizontal polarization for ($\left|{T}_{xx}\right|$) or in vertical polarization for ($\left|{T}_{yy}\right|$). Similarly, for measuring the magnitude of the transmission cross-component ($\left|{T}_{yx}\right|$) and ($\left|{T}_{xy}\right|$), the transmitting and receiving antennas were oriented in a cross-polarized manner. This involved placing the transmitting antenna in horizontal polarization and the receiving antenna in vertical polarization, or vice versa. Figure 11 presents a comparison between the simulated and measured magnitudes of the transmitted components for both x- and y-polarized waves, propagating in both the forward (+ z) and backward (− z) directions across different frequencies. The results clearly indicate a substantial agreement between the measured and simulated data, although minor discrepancies are attributed to misalignment of the horn antennas and environmental noise. Finally, to evaluate the accomplishment of the proposed chiral metasurface, a comparative analysis is conducted against state-of-the-art published work, as summarized in Table 2. The comparison highlights a notable superiority of the proposed chiral metasurface over other existing counterparts.

**Table 2:** Comparison with state-of-the-art multifunctional chiral metasurface polarizers with asymmetric transmission

Ref. No.	Cell size (mm^2^)	Material type	Metallic layers	No. of Bands		Frequency (GHz)		CD
				LP to CP	LP to LP	LP to CP (PER ≥ ± 20 dB)	LP to LP (PCR ≥ 0.9)	
[10]	5 × 5	FR-4	2	2	0	11.65, 13.02	0	N/A
[15]	15 × 15	FR-4	2	2	0	5.1, 6.4	0	N/A
[16]	11.5 × 11.5	Arlon AD250	2	4	0	12.25, 13.9, 15.57, 16.86	0	N/A
[20]	6.6 × 6.6	F4B	2	3	0	8.72, 9.77, 11.84	0	N/A
[21]	5.52 × 5.52	F4B	2	2	0	8.08, 9.94	0	N/A
[22]	10 × 10	FR-4	2	2	1	5.32, 6.6	10.52	N/A
[23]	10 × 10	FR-4	2	2	0	7.8, 10.1	0	N/A
[24]	3.5 × 3.5	FR-4	3	4	0	7.28, 13.22, 15.49, 9.48	0	N/A
[25]	10 × 10	Rogers DiClad 880	2	1	1	14.79	9.15	N/A
[36]	4 × 4	F4B	2	2	0	9.64	0	N/A
[37]	4 × 4	F4B-2	3	3	0	20.2, 47.9	0	N/A
[38]	10 × 10	Rogers RT5870	2	4	0	5.18‒5.23, 10.64‒10.82, 12.25‒12.47, 14.42‒14.67	0	N/A
[39]	12 × 12	Arlon AD-430	2	1	5	4.05‒4.23	8.9–10.7, 12.43–13.25	N/A
[40]	6 × 6	FR-4	2	2	1	7.18‒8.85	15‒17	N/A
Proposed	14 × 14	FR-4	2	3	1	5.013‒5.066, 6.227‒6.297, 16.18‒16.285	14.988‒ 15.634	0.64, 0.74, 0.5

*n/a* not available

## Conclusion

The study introduces a chiral metasurface based polarizer designed to operate across multiple frequency bands and offer diverse functionalities with asymmetric transmission. The metasurface was designed using a 2 × 2 supercell on cost-effective FR-4 material. Within each element, two distinct resonating elements were incorporated. Notably, on the front side of the substrate, the elements were oriented with a 90° clockwise rotation relative to each other, and the right diagonal elements were intentionally scaled down, introducing an anisotropic feature to the structure. The intentional absence of C4 symmetry in the design contributed to the attainment of high cross polarization conversion. Additionally, on the back side of the substrate, the elements were rotated by 90° in comparison to the front side, enhancing the overall chirality of the structure. The proposed chiral metasurface exhibits the capacity to convert incident y-polarized LP wave into RHCP and LHCP waves within the frequency ranges of 5.013‒5.066 GHz and 6.227‒6.297 GHz, respectively. Additionally, for the incident x-polarized wave, it transforms the LP wave into RHCP within the frequency range of 16.18‒16.285 GHz. Furthermore, the metasurface acts as a versatile 90° polarization rotator, converting x-polarized wave into y-polarized wave across a wideband spectrum of 14.988‒15.634 GHz. The efficacy of this chiral metasurface polarizer was assessed using various essential performance parameters and verified through experimental measurement results.

## Data Availability

The datasets generated during and/or analyzed during the current study are available from the corresponding author on reasonable request.
